# The Anti-Cancer Role of Pterostilbene in Endometrial Cancer: A Phase II Prospective, Randomized, Window-of-Opportunity Clinical Trial with Megestrol Acetate

**DOI:** 10.3390/antiox14030345

**Published:** 2025-03-14

**Authors:** Rosemary N. Senguttuvan, Hyejin Cho, Xiwei Wu, Paul H. Frankel, Nora Ruel, Susan E. Yost, Mehdi Kebria, Ernest Han, Mihae Song, Maria de Leon, Marta Invernizzi, Melissa Eng, Raechelle Tinsley, Behrouz Salehian, Aimin Li, Daniel Schmolze, Sue Chang, Javier Arias-Stella, Thanh H. Dellinger

**Affiliations:** 1 Department of Surgery, City of Hope National Medical Center, Duarte, CA 91010, USA; rsenguttuvan@coh.org (R.N.S.); mkebria@coh.org (M.K.); ehan@coh.org (E.H.); misong@coh.org (M.S.); mdeleon@coh.org (M.d.L.); minvernizzi@coh.org (M.I.); 2 Integrative Genomics Core, City of Hope National Medical Center, Duarte, CA 91010, USA; hycho@coh.org (H.C.); xwu@coh.org (X.W.); 3 Department of Computation and Quantitative Medicine, City of Hope National Medical Center, Duarte, CA 91010, USA; pfrankel@coh.org (P.H.F.); nruel@coh.org (N.R.); 4 Department of Medical Oncology, City of Hope National Medical Center, Duarte, CA 91010, USA; suyost@coh.org; 5 Clinical Trials Office, City of Hope National Medical Center, Duarte, CA 91010, USA; meng@coh.org (M.E.); rtinsley@coh.org (R.T.); 6 Department of Endocrinology, City of Hope National Medical Center, Duarte, CA 91010, USA; bsalehian@coh.org; 7 Department of Pathology, City of Hope National Medical Center, Duarte, CA 91010, USA; aili@coh.org (A.L.); dschmolze@coh.org (D.S.); suchang@coh.org (S.C.); jariasstella@coh.org (J.A.-S.)

**Keywords:** megestrol acetate, pterostilbene, endometrial cancer, clinical trial

## Abstract

Pterostilbene (3,5-dimethoxy-40-hydroxystilbene) is a potent oral antioxidant with a promising role in anti-cancer treatment. In endometrial cancer (EC), in vitro studies demonstrated a synergistic antiproliferative effect of pterostilbene (PT) with megestrol acetate (MA), a common treatment for EC. This is a randomized phase II clinical trial (NCT03671811) of PT+MA vs. MA for three weeks prior to scheduled hysterectomy. The primary objective is to determine the antiproliferative effect of PT+MA vs. MA using Ki-67 index. The secondary objectives are toxicity, histological response, transcriptional changes, and lipid metabolism. A total of 44 patients were enrolled between January 2019 and November 2022 with 23 randomized to Arm 1 (PT+MA) and 21 to Arm 2 (MA). Toxicities included one G3 thromboembolic event (PT+MA) and one G3 hypertension event (MA). Histological responses were high in both arms (>90%). There was no difference in Ki-67 changes, although, when restricted to endometroid subtype, the relative decrease in Ki67 was 33.8% in PT+MA vs. 20.1% in MA alone (*p* = 0.14). Whole transcriptomic gene profiling of samples before and after PT+MA exposure demonstrated the activation of interferon alpha response pathway and suppression of mTORC1 signaling, hypoxia, oxidative phosphorylation, and IL2-STAT5 signaling. Lipid metabolism analyses did not reveal any significant changes between arms. PT is well-tolerated in the preoperative treatment of EC and demonstrated in vivo anti-cancer effects on the transcriptomic level.

## 1. Introduction

Endometrial cancer (EC) is the most common gynecologic malignancy in developed countries, and the fourth most common cancer in women overall, largely due to demographic shifts towards increasing obesity, an aging population, and the disproportionate increase in disease burden among minorities [1,2,3,4]. Early-stage EC may be cured with hysterectomy. However, many young EC patients choose to avoid fertility-ending therapies.

It is well known that estrogen receptor signaling drives tumor development for low-grade endometrial cancer, and that estrogen is a critical regulator of the cell cycle regulatory machinery in cancer cells [5]. In addition, progestins are the most widely used form of endocrine therapy in advanced or recurrent endometrial carcinoma. Megestrol acetate (MA) is a progestin that showed response rate in patients with advanced and recurring EC [6,7]. Progesterone therapy counteracts the estrogenic tumor environment and is currently approved as a treatment option in women who wish to forgo definitive surgical treatment or in patients who are poor surgical candidates. There have been promising in vitro and in vivo studies investigating natural supplements known to have potent antioxidant properties in synergy with progesterone therapy [8]. Pterostilbene (3,5-dimethoxy-40-hydroxystilbene) is an antioxidant compound that is found in high concentration in blueberries. It is biochemically similar to resveratrol, a compound found in red wine with similar antioxidant function but demonstrates 80% oral bioavailability compared to resveratrol’s 20% oral bioavailability [9]. It has been shown to improve hyperlipidemia, cardiovascular disease risk, diabetes mellitus, fatty liver, and other diseases that cause dysregulation of metabolic processes [10,11]. Pterostilbene (PT) has shown promising anticarcinogenic and anti-inflammatory results on a biochemical level in multiple types of cancer: breast [12,13], gastric [14], colon [15], pancreatic [16], and endometrial [8,17] to name a few. It has diverse pharmacological activities, including attenuation of adipogenesis as demonstrated through its in vitro regulation of adipogenesis markers [18]. As endometrial cancer is an obesity driven cancer, such role would be beneficial in this cancer. Pterostilbene is also known to reduce tumor cell expansion and apoptosis by influencing the PI3K/Akt MAPK pathways [19,20], which are often dysregulated in endometrial cancer, and are attractive drug targets. We and others have demonstrated that pterostilbene effectively suppresses phosphorylation of STAT3, as well as STAT3 downstream genes that regulate cell cycle and apoptosis. These in vitro studies suggest that pterostilbene facilitates significant anti-tumor activity via anti-proliferative and pro-apoptotic mechanisms, possibly via downregulation of JAK/STAT3 pathway [21]. Our previous investigation in EC suggests the synergy of MA and PT in EC via the suppression of STAT3 and ERK1/2 pathways, along with the modulation of estrogen receptor (ER) expression [8]. The translation of these preclinical findings into animal studies and clinical trials has yet to be sufficiently explored.

The primary objective of this study is to evaluate the in vivo antiproliferative impact of PT in EC patients. Therefore, the primary aim of this study is to demonstrate biochemical efficacy of PT as measured by the Ki-67 index during the preoperative window in patients with EC when given in conjunction with MA. Secondary objectives include safety and tolerability, histologic response, and transcriptomic gene expression changes to determine safety and tolerability in endometrial cancer patients. Histological responses as measured by GOG-211 criteria (glandular secretory change, involution, or atrophy with loss of mitotic activity, and stromal decidualization) were evaluated. We hypothesized that PT may improve the antiproliferative effect of MA by a reduction in antigen Kiel 67 (Ki-67) proliferation index in post-treatment hysterectomy samples compared to pre-treatment endometrial samples. Additionally, we set out to evaluate the in vivo molecular mechanisms of PT+MA, to further evaluate preclinical investigations.

## 2. Materials and Methods

This open-label single institutional phase II trial was conducted between January 2019 and November 2022 with institutional review board (IRB) approval at the City of Hope National Cancer Center (IRB 17327). The trial was conducted in accordance with the World Medical Association Declaration of Helsinki, International Conference on Harmonization Good Clinical Practice guidelines, and the U.S. Code of Federal Regulations. All patients completed informed consent prior to study entry, and the trial was conducted under an open United States clinical trial (NCT03671811).

**Study Design:** The planned accrual for this study was 36 patients (Arm 1, *n* = 18 and Arm 2, *n* = 18). Patients were randomized in a 1:1 ratio to either Arm 1 (oral PT+MA twice a day for three weeks), or Arm 2 (oral MA twice a day for three weeks). Therapy continued in the absence of unacceptable toxicity or progression of disease.

**Eligibility Criteria:** Women aged at least 18 years of age were considered eligible for this trial if they had a preoperative diagnosis of endometrial intraepithelial neoplasia (EIN), complex endometrial hyperplasia, or endometrial cancer of any histologic subtype. EIN represents a continuum with endometrial cancer; up to 22% of patients with EIN have endometrial cancer following hysterectomy, thus patients with EIN were included in this trial. An Eastern Cooperative Oncology Group (ECOG) performance status of ≤2 was required. Exclusion criteria included patients who were not deemed surgical candidates, receipt of prior therapy, phytochemical substances within 30 days prior to protocol therapy, and serious medical diseases.

**Toxicity by CTCAE 4.0:** Adverse events (AEs) were reported according to CTCAE v.4.0 grading system. Patients underwent clinical monitoring immediately after treatment completion and 6 weeks post-treatment.

**Sample collection:** Pre-treatment endometrial biopsies were obtained from patients by office endometrial suction pipelle or via dilation and curettage in the operating room. Patients who had their pathological diagnosis outside of our institution had their pathology overread at our institution. Patients then underwent approximately 3 weeks of treatment therapy, following which they underwent standard of care hysterectomy. Formalin-fixed paraffin-embedded (FFPE) tissues from both pre- and post-treatment specimens were prepared according to standard procedures.

**Immunohistochemical (IHC) staining using Ki-67 antibody:** FFPE blocks were sectioned, and tissue slides were de-paraffinized, hydrated, and incubated with the antibody. Analysis was performed according to standard procedures (dilution ratio 1:100, antibody MIB-1, pharmDx Dako Omnis assay). The Ki-67 index was measured via hot spot score (average percentage across hot-spot areas of proliferation) and global score (average percentage across different tumor areas with different areas of proliferation) in the pre-treatment tumor and post-treatment hysterectomy specimen by three blinded pathologists (S.C., D.S., and J.A.S). The number of positive staining nuclei irrespective of staining intensity was scored through visual estimation of at least three high-power fields (x40 magnification) across whole tumor tissue sections, expressing the Ki-67 score as the percentage of positively stained cells among the total number of tumor cells evaluated. A final Ki-67 score was calculated as the mean value of the three pathologists’ scores.

**Histological response using GOG-211:** Criteria for histologic response was determined using the GOG-211 cooperative group trial evaluating MA in a preoperative window trial in EC patients [6,7]. GOG-211 defined categorical variables and subjectively categorized complete histologic response, partial histologic response, or no histologic response. A complete histologic response was defined as diffuse glandular secretory change, involution, or atrophy, with loss of mitotic activity, and stromal decidualization. No histologic response was defined as persistent diffuse architectural features of adenocarcinoma. A partial histologic response was defined as a mixture of both. The initial pre-treatment endometrial biopsy specimen was compared to the post-treatment hysterectomy specimen, based on above GOG-211 criteria, blinded between the two arms. The features of histologic response include histologic grade, nuclear grade, nucleoli, nuclear stratification, eosinophilic metaplasia, squamous metaplasia, mucinous metaplasia, secretion, mitotic index, gland cellularity, and decidual change.

**Biomarkers of response by immunohistochemistry (IHC):** Molecular markers to assess cell cycle regulation and cell survival including Cyclin D (CDK4), Cyclin D1, BCL2, phosphorylated STAT3 (p-STAT3), and phosphorylated ERK1/2 (p-ERK1/2). Other IHC markers to evaluate progression/regression included estrogen receptor (ER) and progesterone receptor (PR). Biomarkers of response were analyzed at the beginning and completion of treatment.

**Transcriptional analysis:** Changes in RNA expression were analyzed at the beginning and at completion of treatment. RNA was isolated by the molecular pathology core at COH and at TGen according to standard procedures (10 unbaked slides at 10 μm), and whole transcriptome sequencing was by TGen. Bioinformatics analysis was performed by the Integrative Genomics Core (IGC) at City of Hope, under the supervision of Dr Xiwei Wu. Adapters in raw RNA sequenced reads were trimmed using Trimmomatic [22] and poly(A) tails were removed using FASTP [23] STAR aligner [24] with default settings was used to align the trimmed reads to human genome hg38. Raw counts of RefSeq genes were acquired using “Rsubread” R package Version 2.8.2 [25]. Normalization of the raw counts were achieved by trimmed mean of M-values (TMM) method in “edgeR” Bioconductor package Version 3.36.0 [26]. Genes with RPKM ≥ 1 in at least two samples remained for differential expression analysis using the limma-trend approach in “limma” R limma package Version 3.50.3 [25]. The differentially expressed genes were selected based on the cutoff values *p*-value < 0.05 and fold change > 1.5. The gene set enrichment analysis (GSEA) was performed using GSEA software v. 4.3.2 [27] to identify the affected Hallmark pathways from msigdbr R package Version 7.5.1 [28], using the pre-ranked gene list ranked by the −log10 (*p*-value) with a sign determined by the fold change direction.

**Laboratory and physical evaluations:** Lipid panel (HDL, LDL, triglycerides), glucose, CBC, serum chemistry, weight, and BMI were collected at baseline, following treatment completion, and at 6 weeks following treatment completion.

**Statistical Analysis:** The planned sample size was 36 total evaluable participants, with 18 per treatment arm. With 18 evaluable subjects per arm and using a 1-sided *t*-test at *p* < 0.05, the trial was intended to provide 90% power to detect a difference in efficacy, favoring MA plus PT, of at least 10% (with standard deviation 10%) in the mean reduction in Ki-67. Randomization was 1:1, allocated within strata of diagnosis and menopausal status. All patients who received at least one dose of protocol therapy were evaluable for toxicity. Subjects who received at least 10 days of study treatment and provided pre- and post-treatment tissue samples were evaluable for the efficacy endpoint using Ki-67. Non-evaluable subjects who received at least 1 dose of study treatment were included in the toxicity analysis but were excluded from the efficacy (Ki-67) analysis. Patient demographics and clinical characteristics at baseline were summarized using descriptive statistics.

## 3. Results

This is a randomized phase II clinical trial (NCT03671811) for EC patients. The primary objective is to determine the effect of PT+MA versus MA using Ki-67 index. Secondary objectives are toxicity, proliferation by Ki-67 index, histological response, biomarkers of response, transcriptional changes, and lipid profile.

### 3.1. Phase II Clinical Trial

#### 3.1.1. Study Design and Enrollment

**Figure 1:**
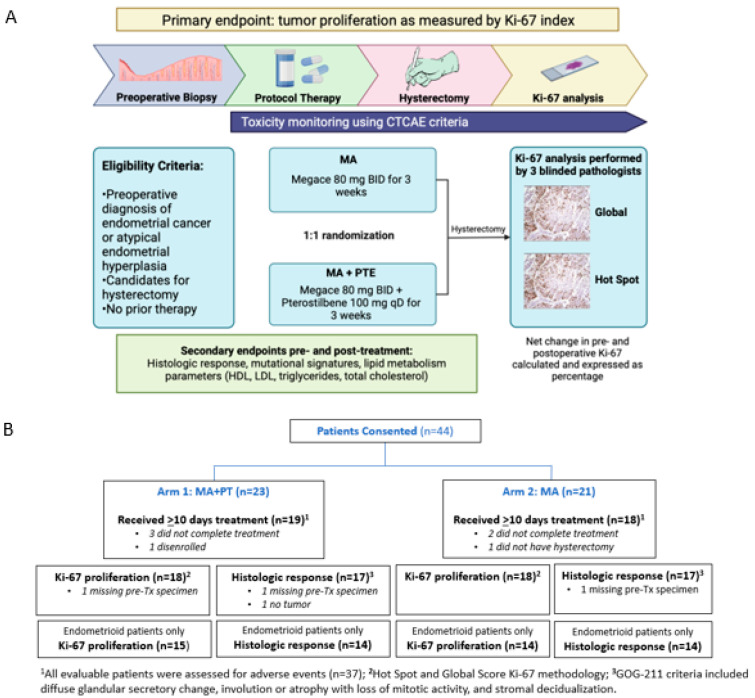
(**A**) Study schema of phase II randomized 2-arm study with primary endpoint Ki-67 index. Baseline endometrial biopsy was obtained as block or slides from initial diagnosis, or as new office endometrial biopsy. Intra-operative endometrial biopsy at time of hysterectomy was obtained if feasible. Post-treatment endometrial tumor was also obtained as block/slides if surgery was performed at a hospital outside of COH. (**B**) Flow diagram showing patients included in the study grouped by Arm 1 (*n* = 19) and Arm 2 (*n* = 18). Seven patients were not evaluable (5 received <10 days of treatment, 1 did not have hysterectomy, and 1 disenrolled). AE, adverse event; MA, megestrol acetate; PT, pterostilbene; Tx, treatment.

A total of 207 patients with EC were screened across four community network sites at our institution. A total of 44 patients were enrolled in this study between January 2019 and November 2022. Additionally, 23 patients were randomized to Arm 1 (PT+MA), and 21 patients were randomized to Arm 2 (MA), of which 37 patients were evaluable (5 received <10 days of study treatment, 1 disenrolled, and 1 did not receive hysterectomy). The study schema and flow diagram are shown in Figure 1.

#### 3.1.2. Patient Characteristics

Baseline patient characteristics are listed in Table 1. Median age was 61.9 years (57.3–70.9); 62% were non-Hispanic white and 38% Hispanic. Baseline demographics were comparable between both arms. Median treatment duration was 21 days (14–56) and was similar in both arms.

### 3.2. Toxicity

All 37 evaluable patients were assessed for adverse events, including 19 in Arm 1 and 18 in Arm 2. Adverse events (Grade 2 and 3) attributed to study drugs (PT+MA or MA) or surgery per CTCAE 4.0 are shown in Table 2 (*n* = 37). One G3 thromboembolic event was reported in Arm 1 (PT+MA), and one G3 hypertension in Arm 2 (MA).

### 3.3. Proliferation by Ki-67 Index

**Figure 2:**
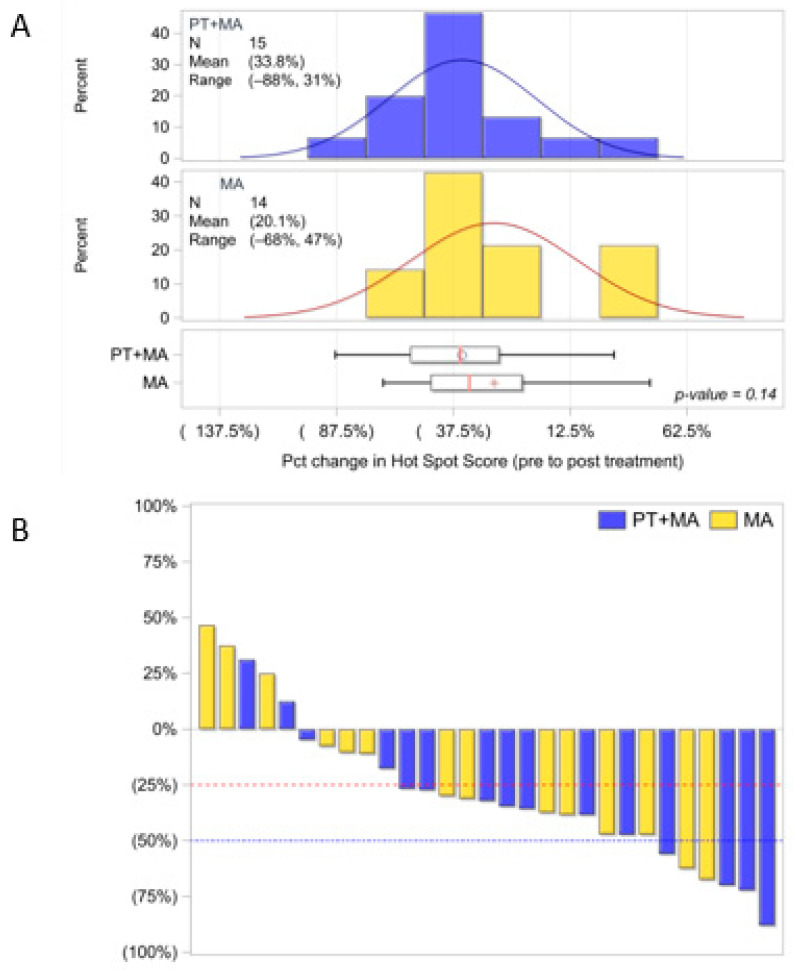
Evaluable endometrioid patients Ki-67 distribution utilizing hot spot score methodology by three pathologists (PT+MA, *n* = 15; MA, *n* = 14). (**A**) Ki-67 distribution of hot spot mean score for PT+MA is −33.8% and MA mean score is −20.1%; *p* = 0.14; (**B**) Waterfall plot for percent decrease in Ki-67 from pre-Tx to post-Tx [(post−pre)/pre]. Parentheses around the mean percentages represent a decrease (negative). The circle and + symbols in the boxes represent the group mean. Percent, %; Treatment, Tx; Pct, percent (1:100, Dako, MIB-1).

Of the 37 evaluable patients, Ki-67 analysis was performed for 18 in Arm 1 (1 missing pre-treatment specimen) and 18 in Arm 2 (*n* = 36). The hot spot score for Ki-67 analysis showed standard deviations were not significantly different between pre-treatment and post-treatment values. As primarily EC patients of endometrioid histology respond to hormonal therapy, we also analyzed the subcohort of 29 patients with G1+G2 endometrioid histology (Arm 1, *n* = 15 and Arm 2, *n* = 14). The distribution of hot spot scoring amongst the three pathologists is shown in Figure 2A, and the percent change (between pre- and post-treatment Ki-67 indices) is demonstrated by the waterfall plot in Figure 2B. The endometrioid PT+MA group demonstrated a larger decrease in Ki-67 with a -33.8% change compared to −20.1% in the MA group, which was not statistically significant (*p* = 0.14, one-sided).

**Figure A1:**
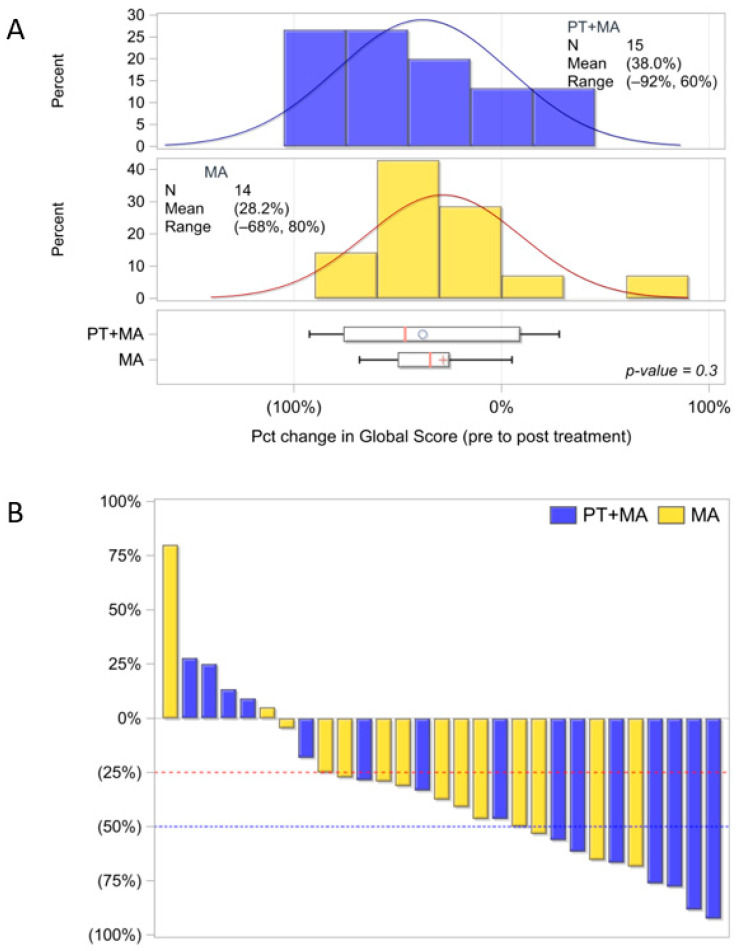
Evaluable endometrioid patients Ki-67 distribution utilizing global score methodology by three pathologists (PT+MA, *n* = 15; MA, *n* = 14). (**A**) Ki-67 distribution of global mean score for PT+MA is −38.0% and MA mean score is −28.2%, *p* = 0.3; (**B**) Waterfall plot for % decrease in Ki-67 from pre-Tx to post-Tx [(post−pre)/pre]. Parentheses around the mean percentages represent a decrease (negative). The circle and + symbols in the boxes represent the group mean. Percent, %; Treatment, Tx; Pct, percent (1:100, Dako, MIB-1).

**Figure A2:**
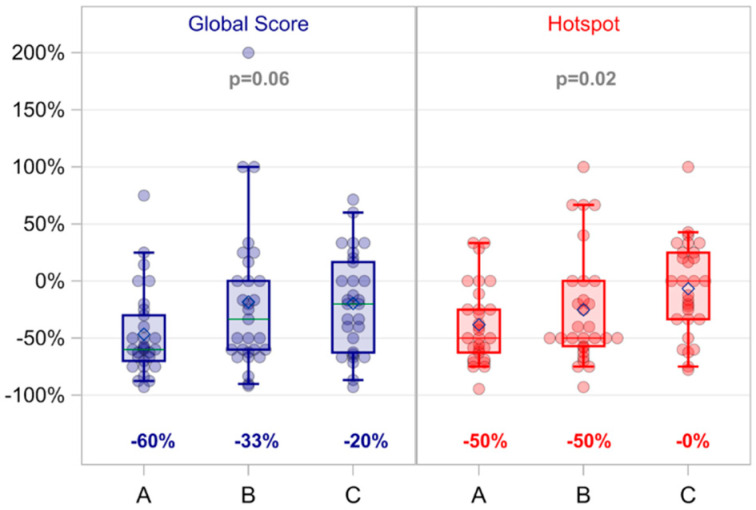
Global score and hot spot differences by pathologist. Each score was summarized as the average of three blinded pathologists. Global score, *p* = 0.06; Hot spot score, *p* = 0.02.

Global scoring Ki-67 proliferation PT+MA (*n* = 18) and MA (*n* = 18) also reported standard deviations were not significantly different between pre-treatment and post-treatment values. Similarly, the subcohort of 29 patients with G1+G2 endometrioid histology (PT+MA, *n* = 15 and MA, *n* = 14) did not demonstrate significant differences between arms (Figure A1A). The global score for endometrioid patients was -38.0% and MA score −28.2%; *p* = 0.25 [(post−pre)/pre]. The change (between pre- and post-treatment Ki-67 indices) is demonstrated by the waterfall plot (Figure A1B). Interpathologist variability was high for all methods of Ki-67 scoring and likely contributed to lack of homogeneous results (Figure A2).

### 3.4. Histological Response Using GOG-211 Criteria

**Figure 3:**
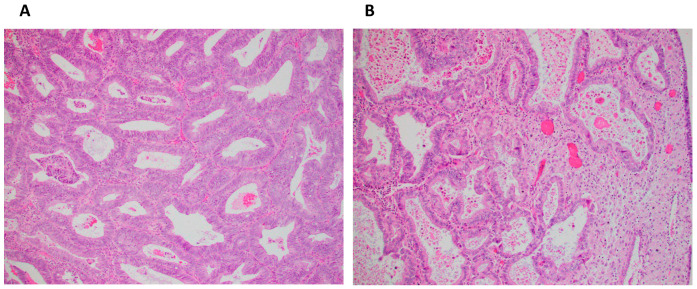
(**A**) Pretreatment biopsy sample with endometrial adenocarcinoma, endometrioid type, FIGO grade 1 (×200); (**B**) Post-treatment hysterectomy sample of same patient with partial histologic response characterized by loss of nuclear stratification, decrease in glandular cellularity, acquisition of abundant eosinophilic cytoplasm, occasional luminal secretion, stromal decidual change, and focal atrophic glands (×200).

Histological response was determined for 17 in Arm 1 (1 missing pre-treatment specimen, 1 no tumor) and 17 in Arm 2 (1 missing pre-treatment specimen). GOG-211 criteria [6,7] included diffuse glandular secretory change, involution, or atrophy with loss of mitotic activity, and stromal decidualization from pre- to post-treatment in all patients (Table A1). Histological response was high in both arms (>90%), likely contributing to lack of statistical significance between arms. A subset of endometrial patients only (Arm 1, *n* = 14 and Arm 2, *n* = 14) did not reveal any differences between arms. Additional analysis of histologic changes including any improvement in (1) histologic grade, (2) nuclear grade, (3) nucleoli, (4) mitotic index, or (5) glandular cellularity from pre- to post-treatment in all patients (Arm 1, *n* = 17 and Arm 2, *n* = 17), and endometrial patients only (Arm 1, *n* = 14 and Arm 2, *n* = 14) also did not show a difference between arms. There were some instances of histological response in individual cases in both arms. Figure 3 demonstrates a pre- and post-treatment example of a patient in the PT+MA arm that showed a response to therapy.

### 3.5. Biomarkers of Response by IHC

Molecular markers of all eligible patients (*n* = 37) were analyzed at the beginning and completion of treatment to evaluate cell cycle regulation and cell survival (CDK4, Cyclin D1, BCL2, p-STAT3, and p-ERK1/2) and progression/regression (ER, PR). The results showed no significant differences between Arm 1 (*n* = 19) and Arm 2 (*n* = 18).

### 3.6. Transcriptional Analysis

Changes in RNA expression were analyzed at the beginning and completion of treatment. The paired analysis was performed by comparing individual patient’s post-treatment sample to their pre-treatment sample. The difference in each paired sample was then compared to the other paired samples in each arm to identify differentially expressed genes, and gene set enrichment analysis was performed.

**Figure 4:**
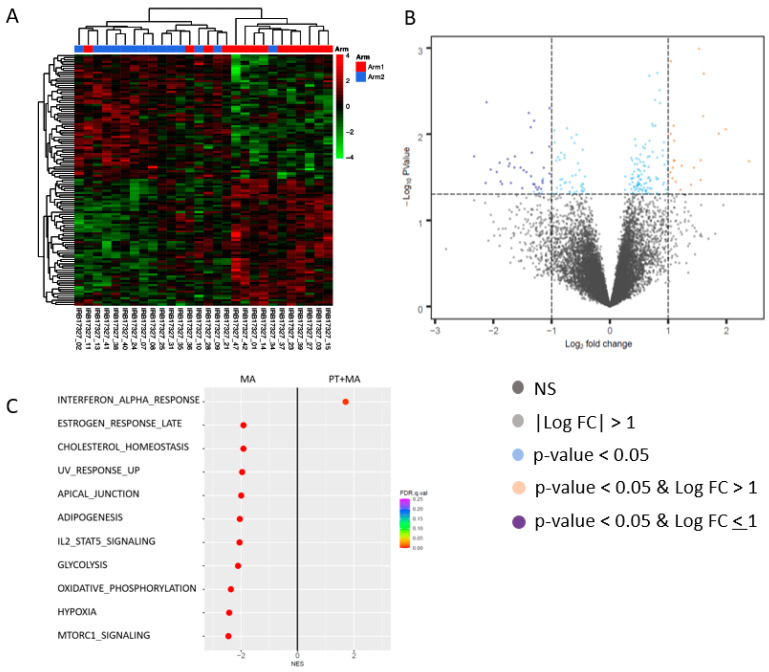
Differentially expressed genes in endometrioid patients for Arm 1 (PT+MA) and Arm 2 (MA). This paired analysis was performed by comparing individual patient’s post-treatment sample to their pre-treatment sample. The difference in each paired sample was then compared to the other paired samples in each arm. (**A**) Hierarchical cluster analyses of pre-treatment and post- treatment tumor samples in each arm identified differentially expressed genes, and the differential gene expression of these “post−pre” changes were compared between arms (samples are not clustered by arm); (**B**) Among endometrioid patients, tumor analysis resulted in 117 differentially expressed genes between the arms (fold-change > 1.5, *p* < 0.05); (**C**) Hallmark GSEA results for PT+MA (post–pre)-MA (post–pre). Hallmark_Interferon_Alpha_Response (M5911), Hallmark_MTORC1_Signaling (M5924), Hallmark_Hypoxia (M5891), Hallmark_Oxidative_Phosphorylation (M5936), Hallmark_Il2_Stat5_Signaling (M5947). Among PT+MA responders, significant molecular changes include upregulation of interferon alpha response pathway, and downregulation of mTORC1 signaling, hypoxia, oxidative phosphorylation, and IL2-STAT5 signaling. NES, normalized enrichment score; FDR, false discovery rate.

Differentially expressed genes were selected based on the cutoff values *p*-value < 0.05 and fold change > 1.5, and gene set enrichment analysis was performed to identify the affected Hallmark pathways using the pre-ranked gene list ranked by the −log10 (*p*-value) with a sign determined by the fold change direction. Hierarchical cluster analyses of pre-and post-treatment tumor samples in each arm identified differentially expressed genes (Arm 1, *n* = 17; Arm 2, *n* = 15) (Figure 4A). Gene expression changes in each arm between post-and pre-treatment samples were obtained. The differential gene expression of these “post−pre” changes were then compared between arms. Among endometrioid tumors, comparison of gene expression between PT+MA versus MA alone resulted in 117 differentially expressed genes between the arms (fold-change > 1.5, *p* < 0.05). Among statistically significantly upregulated and downregulated genes, no genes demonstrated a significant association with known PT mechanistic effects (Figure 4B).

The comparison of enriched Hallmark pathways between PT+MA versus MA arms alone was performed to determine the molecular effects of PT. Significantly enriched pathways in the PT+MA arm compared to MA only arm represent pathways activated by PT. Conversely, enriched pathways in the MA alone pathway as compared to PT+MA were interpreted as pathways suppressed by PT. The most significant molecular changes affected by the addition of PT was the activation of the interferon-alpha response pathway (Figure 4C). The comparison of GSEA in MA-treated tumors to PT+MA-treated tumors demonstrated pathways suppressed by PT, and included important pathways central to tumor progression, including oxidative phosphorylation, glycolysis, hypoxia, and mTOR signaling. This represents a significant role of PT in restraining these pro-tumorigenic pathways. Additional anti-cancer pathways suppressed by PT (i.e., enriched in MA alone) included adipogenesis, IL2/STAT5, cholesterol homeostasis, estrogen response late, UV radiation upregulated genes, and apical junction.

### 3.7. Laboratory and Physical Evaluations

**Figure A3:**
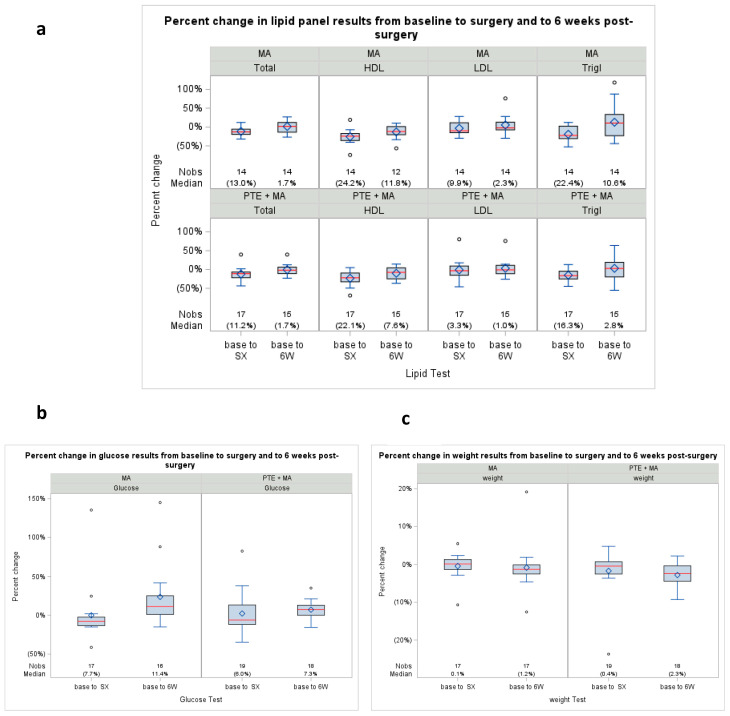
Percent change in lipid panel results from baseline, surgery, and 6 weeks post-surgery. (**a**) Change in lipid panel measurements pre- to post-Tx (baseline to surgery, and baseline to 6 weeks) measured in % difference; (**b**) Change in glucose from baseline pre- to post-Tx measured in % difference; (**c**) Change in weight from baseline pre- to post-Tx measured in % difference. Parenthesis around a single percentage represents a decrease (negative). All *p*-values were ≥0.5. Percent, %; Treatment, Tx.

The lipid panel (HDL, LDL, triglycerides), glucose, CBC, serum chemistry, weight, and BMI were collected at baseline, following treatment completion, and at 6 weeks following treatment completion. The evaluation of parameters related to lipid metabolism revealed no significant differences between pre- and post-treatment when analyzed in both arms (Arm 1, *n* = 17 and Arm 2, *n* = 16). Detailed data regarding pre- and post-treatment lipid profiles, glucose measurements, and weight are reported in Figure A3.

## 4. Discussion

Pterostilbene is a commercially available natural supplement that is commonly marketed for health prevention with anti-inflammatory and antioxidant qualities. While heralded as potential anti-cancer agent with extensive pre-clinical evidence, limited clinical studies exist to establish an anti-cancer role. As patients and consumers veer towards natural preventative supplements in precancer and early oncologic diseases, the role of dietary supplements ought to be further investigated.

In this window-of-opportunity study, we sought to determine if the addition of PT to conventional MA therapy could meaningfully reduce the rate of proliferation when given preoperatively in patients with newly diagnosed endometrial adenocarcinoma. This hypothesis was based on promising preclinical data indicating a synergy between PT+MA. Additionally, we aimed to affirm the safety and tolerability of PT.

Overall, female endometrial cancer patients who were given oral daily pterostilbene, tolerated this administration well over a three-week course. Severe adverse events (AE) including one grade 3 venous thromboembolic event (VTE) are most likely attributed to megestrol acetate, which was given concurrently with pterostilbene, and is known to increase risks of VTE, though could also be attributed to postoperative surgical sequelae from hysterectomy. Nonetheless, the combination of both agents makes it difficult to distinguish causality of toxicities. Given the clinical demographics and medical comorbidities in patients with newly diagnosed endometrial cancer often overlap with the medical comorbidities commonly seen in patients with obesity and insulin resistance, the grade 3 AEs noted may be secondary to medical/surgical intervention in a patient population already at a baseline elevated risk. Overall, no AEs or toxicities were attributed to the addition of PT. Regardless, PT appears to be a well-tolerated addition to the preoperative medication regimen.

The addition of pterostilbene suggested greater reduction in the cancer growth rate measure, Ki-67 index, in endometrial samples than compared with MA treatment alone, though did not reach statistical significance. Similar results of a neuroblastoma cell line treated with pterostilbene did not show statistically significant variations [29]. Ki-67 is a measure of the rate of cancer cell division; thus, a higher rate indicates more aggressive cancer growth. Endometrioid patients treated with PT and MA associated with a Ki-67 decrease of 33.8%, while in patients treated with megestrol acetate alone, the Ki-67 shrunk by only 20.1%. The difference between these two groups did not reach statistical significance (*p* = 0.14). Despite this, some benefits appear to be derived at a histological level given the >10% reduction in Ki-67 scoring in the PT+MA arm as compared to the control arm in Ki-67 index using two different scoring modalities (global and hot spot scoring). Future studies are needed to evaluate the cancer preventative role of PT outside of the combination with MA, including as monotherapy or in combination with other drugs. In endometrial cancer, combination with anti-obesity drugs may be relevant due to obesity presenting a major risk factor for the development of this cancer. Additionally, biomarker studies can help determine more effective roles for PT.

Another measure of response is to evaluate histologic response through a number of microscopic tissue comparisons, including diffuse glandular secretory change, involution or atrophy, loss of mitotic activity, and stromal decidualization from pre- to post-treatment endometrial samples, as established by a national cooperative group study (GOG 211) [6,7]. The histologic response for all endometrial samples in this group was high, at greater than 90%. No difference was demonstrated between the two arms, likely due to the high histologic response rate of >90% possibly masking any significant difference between the two arms.

We evaluated the downstream molecular effects of pterostilbene addition to megestrol acetate in endometrial cancer.

Pterostilbene significantly suppressed many important pathways central to tumor progression, including oxidative phosphorylation, glycolysis, hypoxia, and mTOR signaling.

Oxidative phosphorylation is an active cancer pathway upregulated in tumors and cancer stem cells and is significantly suppressed in endometrial cancer tissues of patients administered pterostilbene. Hypoxia, another fundamentally important feature of solid tumors, was similarly reduced in EC patient tumors upon oral pterostilbene administration.

The mTOR pathway is frequently activated in tumors, and an attractive pathway for novel drug targets in endometrial cancer. Based on preclinical studies, pterostilbene is known to reduce tumor cell expansion and apoptosis by influencing the PI3K/Akt MAPK pathways [19,20]. The current study is the first in vivo study to demonstrate pterostilbene’s role in suppressing the mTORC1 pathway in human endometrial cancer samples.

Endometrial cancer development is marked by obesity, its most predominant risk factor. In this study, pterostilbene significantly reduced adipogenesis in endometrial tissues, thus possibly contributing to reduced cancer metabolism. As pterostilbene’s role in attenuation of adipogenesis was demonstrated through its in vitro regulation of adipogenesis markers [18], our study contributes to the field by confirming pterostilbene’s anti-adipogenic effects in human endometrial cancer.

The IL2/STAT5 signaling pathway plays a critical role in modifying the functional activity of regulatory T cells [30]. We and others have demonstrated that pterostilbene effectively suppresses phosphorylation of STAT3, as well as STAT3 downstream genes that regulate cell cycle and apoptosis. These in vitro studies suggest that pterostilbene facilitates significant anti-tumor activity via anti-proliferative and pro-apoptotic mechanisms, possibly via downregulation of JAK/STAT3 pathway [21]. In our current study, we confirm the preclinical findings of IL2-STAT5 pathway downregulation in human endometrial cancer via whole transcriptome sequencing analysis.

The role of cholesterol metabolism in cancer is well established. Dyshomeostasis of cholesterol is one of the hallmarks of cancer. In cancer cells, cholesterol uptake and synthesis rates are usually increased [31], leading to abnormal metabolism. In endometrial cancer, cholesterol homeostasis-related gene signatures have been demonstrated to correlate with prognosis of this cancer [32]. Indeed, in our study, pterostilbene reduces cholesterol homeostasis, and is likely restraining dyshomeostasis and abnormal cancer-related metabolism.

Endometrial cancer is a hormonally driven cancer marked by estrogen receptor associated proliferation. Estrogen receptors represent the key oncogenic signal in many endometrial cancers [5]. While anti-estrogen agents such as megestrol acetate are commonly used in endometrial cancer treatment, the combination of PT and MA may have a higher inhibitory effect on ER-alpha expression in vitro [21]. It is thus highly relevant that our transcriptomic data reveal that the combination of PT and MA demonstrates significant inhibition of the Estrogen Response Signaling pathway.

Additional significantly downregulated gene signatures include the UV Radiation Upregulated Genes pathway, a pathway rich in upregulated DNA damage and inflammatory genes, and thus likely indicates downregulation of these genes upon pterostilbene treatment.

On the other hand, pterostilbene is known to enhance innate immune activation by increasing IFN-α and IFN-β expression, as demonstrated by in vitro studies in respiratory virus infected cells [33]. We have demonstrated that interferon-alpha is the most activated signaling pathway in endometrial tumors treated by pterostilbene. As interferon-alpha signaling plays a critical role in anti-cancer regulation, this mechanism by pterostilbene contributes significantly to its arsenal of anti-tumor effects.

Our differential molecular data reveal that PT+MA is associated with molecular signatures responsible for upregulation of the interferon-alpha immune response, a critical component in regulation of the tumor microenvironment. The downregulation of MTORC1 is an important benefit noted in our data, as activation of MTORC1 in tumor cells is known to potentiate tumor dissemination and proliferation. STAT5, a potent driver of oncogenesis, is downregulated in patients with a histologic response to PT+MA [28]. As a potent antioxidant, the downregulation in oxidative stress seen in patients with exposure to PT may be intuitive but does validate what is known about the mechanism of the medication.

With obesity as a known driver of EC due to effects of estrogen potentiation, downregulation of downstream genes related to adipogenesis, and other anabolic processes related to cholesterol metabolism were expected along with a concurrent upregulation in genes related to cholesterol homeostasis. This was not uniformly the case in our dataset, though some indicators of PT’s effects on cholesterol regulation were concordant with previously described data. We did note expected downregulation of downstream genes related to late estrogen response, adipogenesis, and oxidative phosphorylation in Hallmark analysis. Our exploratory analysis of pre- vs. post-treatment lipid profiles, glucose values, weight, and BMI between the two arms did not reveal any demonstrable evidence towards PT’s known positive effects on lipid profiling and metabolism, though this may be due to a short treatment duration of only 3 weeks.

The addition of pterostilbene to megestrol acetate preoperatively in patients with endometrial cancer presents a unique window of opportunity study to evaluate the effect of PT in vivo. The toxicities of PT are negligible and there appears the direction of change suggests possible benefit derived on a histological and molecular level.

Limitations of this study include small sample cohort of patients with a short treatment duration. This was a limiting factor given the pathological heterogeneity between cases. Additionally, there was subjectivity in terms of pathologic analysis of histologic response and Ki-67 indices. The optimal method for Ki-67 analysis is not fully agreed upon in the pathology literature and is subject to heterogeneity from pathologist to pathologist increasing the variability. We did initially perform a third Ki-67 scoring methodology (H-score) but given extreme inter-pathologist heterogeneity, this was not used in final analysis. We attempted to mitigate biases with multiple blinded pathologists. Additional limitations include that the pathways analysis was not pre-specified. As a result, both the gene expression and pathway analysis are exploratory and hypothesis generating.

## 5. Conclusions

The addition of PT to MA in the treatment of endometrial cancer patients is well tolerated. Histological response was high in all participants of the study, thus likely mitigating any statistically significant differences in both arms of the trial. Ki-67 as a response outcome is not well established in endometrial cancer and its interpretation is not uniform. When restricted to endometroid subtype the relative decrease in Ki67 was 33.8% in PT+MA vs. 20.1% in MA alone (*p* = 0.14). Our study suggested in vivo anti-cancer effects of pterostilbene, as evidenced by activating the interferon alpha response pathway on whole transcriptome sequencing and confirming pre-clinical studies. The addition of PT to MA also resulted in suppression of numerous pro-tumorigenic pathways, confirming the various roles of PT in downregulation of mTOR signaling, adipogenesis, and IL-2/STAT signaling. As prior investigations demonstrated suppression by PT+MA of STAT3 and ERK1/2 pathways, along with modulation of ER expression, these transcriptomic analyses support the in vivo effects of this combination [8]. In endometrial cancer, the oral antioxidant pterostilbene can be safely added to supplement anti-cancer effects of hormonal treatment. Additional studies are required to evaluate the role of PT in endometrial cancer patients.

## Figures and Tables

**Table 1:** Baseline patient characteristics (*N* = 37).

	Arm 1 (MA+PT) (*n* = 19)	Arm 2 (MA) (*n* = 18)	All Evaluable Patients (*n* = 37)
Median Age (y), (IQR)	61.3 (57.3–70.9)	63.9 (56.7–71.5)	61.9 (57.3–70.9)
Race			
Asian	0 (0.0%)	4 (22.2%)	4 (10.8%)
Black	0 (0.0%)	2 (11.1%)	2 (5.4%)
Pac/Islander	0 (0.0%)	1 (5.6%)	1 (2.7%)
White	17 (89.5%)	10 (55.6%)	27 (73.0%)
Unknown/Not disclosed	2 (10.5%)	1 (5.6%)	3 (8.1%)
Ethnicity			
Hispanic or Latino	8 (42.1%)	6 (33.3%)	14 (37.8%)
Non-Hispanic or Latino	11 (57.9%)	12 (66.7%)	23 (62.2%)
ECOG			
0	8 (42.1%)	8 (44.4%)	16 (43.2%)
1	11 (57.9%)	10 (55.6%)	21 (56.8%)
Menopausal status			
Postmenopausal	18 (94.7%)	17 (94.4%)	35 (94.6%)
Premenopausal	1 (5.3%)	1 (5.6%)	2 (5.4%)
FIGO uterine (path)			
IA	10 (52.6%)	8 (44.4%)	18 (48.6%)
IB	6 (31.6%)	1 (5.6%)	7 (18.9%)
II	1 (5.3%)	4 (22.2%)	5 (13.5%)
IIIA	1 (5.3%)	2 (11.1%)	3 (8.1%)
IIIC1	0 (0.0%)	1 (5.6%)	1 (2.7%)
IVB	0 (0.0%)	1 (5.6%)	1 (2.7%)
Unstaged	1 (5.3%)	0 (0.0%)	1 (2.7%)
Unknown/missing	0 (0.0%)	1 (5.6%)	1 (2.7%)
Path Histology			
Carcinosarcoma	1 (5.3%)	0 (0.0%)	1 (2.7%)
Endometrioid	16 (84.2%)	14 (77.8%)	30 (81.1%)
Endometrioid hyperplasia with atypia	1 (5.3%)	1 (5.6%)	2 (5.4%)
Mucinous	0 (0.0%)	1 (5.6%)	1 (2.7%)
Serous	1 (5.3%)	2 (11.1%)	3 (8.1%)
Pathologic grade			
1	10 (52.6%)	8 (44.4%)	18 (48.6%)
2	6 (31.6%)	7 (38.9%)	13 (35.1%)
3	3 (15.8%)	2 (11.1%)	5 (13.5%)
NA	0 (0.0%)	1 (5.6%)	1 (2.7%)
Cytologic result			
Negative	13 (68.4%)	11 (61.1%)	24 (64.9%)
Not Applicable	5 (26.3%)	6 (33.3%)	11 (29.7%)
Positive	1 (5.3%)	1 (5.6%)	2 (5.4%)
No. pelvic nodes removed			
0	8 (42.1%)	8 (44.4%)	16 (43.2%)
1–2	5 (26.3%)	5 (27.8%)	10 (27.0%)
3–6	4 (21.1%)	3 (16.7%)	7 (18.9%)
NA	2 (10.5%)	2 (11.1%)	4 (10.8%)
Lymphovascular invasion			
Absent	16 (84.2%)	11 (61.1%)	27 (73.0%)
Present	3 (15.8%)	6 (33.3%)	9 (24.3%)
NA	0 (0.0%)	1 (5.6%)	1 (2.7%)
Median Tumor size (cm), (IQR)	3.7 (1.0–5.0)	5.1 (3.0–7.3)	4.2 (2.0–7.0)
Median Treatment Duration (d), (IQR/Range)	20 (16–22/14–56)	21 (20–28/14–52)	21 (18–27/14–56)
<3 weeks	10 (52.6%)	8 (44.4%)	18 (48.6%)
≥3 weeks	9 (47.4%)	10 (55.6%)	17 (52.4%)

**Table 2:** Adverse events (Grade 2 and 3) attributed to study drugs (PT+MA or MA) or surgery per CTCAE 4.0 (*N* = 37).

	Arm 1 (PT+MA) *N* = 19		Arm 2 (MA) *N* = 18		Total *N* = 37	
Adverse Event	Grade 2	Grade 3	Grade 2	Grade 3	Grade 2	Grade 3
Constipation	1				1	
Dizziness			1		1	
Fatigue	1				1	
Gait disturbance			1		1	
Headache	1				1	
Hypertension				1		1
Rash maculo-papular	1				1	
Thromboembolic event		1				1
Urinary tract infection	1				1	
Uterine pain	1				1	

## Data Availability

The original contributions presented in this study are included in the article. Further inquiries can be directed to the corresponding author.

## Appendix A. Data Supplemental to the Main Text

**Table A1:** GOG-211 histological criteria for response rates.

Histological Response Features	Values	Rules	Score
Histologic grade	1, partial; 0, none; 9, uncertain	Partial response, any decrease; Complete response, NA; No response, stays flat or increases	1, 2, 3
Nuclear grade	1, partial; 0, none; 9, uncertain	Partial response, any decrease; Complete response, NA; No response, stays flat or increases	1, 2, 3
Nucleoli	1, partial; 0, none; 9, uncertain	Partial response, present to absent; Response, present to NA; No response, present to present	Absent; Present
Nuclear stratification	1, partial; 0, none; 9, uncertain	Partial response, decrease; Complete response, NA; No response, stays flat or increases	Absent, 0; Mild, 1; Moderate, 2; Marked, 3
Eosinophilic metaplasia	1, partial; 0, none; 9, uncertain	Some response, neg to pos; No response, neg to neg or pos to neg; No tumor content, NA	Negative, <10%Positive, ≥10%
Squamous metaplasia	1, partial; 0, none; 9, uncertain	Some response, neg to pos; No response, neg to neg or pos to neg; No tumor content, NA	Negative, <10%Positive, ≥10%
Mucinous metaplasia	1, partial; 0, none; 9, uncertain	Some response, neg to pos; No response, neg to neg or pos to neg; No tumor content, NA	Negative, <10%Positive, ≥10%
Secretion	1, partial; 0, none; 9, uncertain	Luminal/Subnuclear/Apical, increased secretion; Response, absent to any secretion; No response, any secretion to absent	Luminal, Subnuclear, Apical, Absent
Mitotic index	1, partial; 0, none; 9, uncertain	Some response, decrease; Complete response, NA; Partial response, tumor but no mitosis; No response, non-decreasing	Mitosis per HPF (average of 3 fields)
Gland cellularity	1, partial; 0, none; 9, uncertain	Some response, decrease; Complete response, 0; No response, non-decreasing	Number of cells in 1/4 HPF (average of 3 fields)
Decidual change	1, partial; 0, none; 9, uncertain	Partial response, absent to present; No response, present to absent or present to present	Absent; Present
