# Correction: Senguttuvan et al. The Anti-Cancer Role of Pterostilbene in Endometrial Cancer: A Phase II Prospective, Randomized, Window-of-Opportunity Clinical Trial with Megestrol Acetate. *Antioxidants* 2025, *14*, 345

**DOI:** 10.3390/antiox14121479

**Published:** 2025-12-10

**Authors:** Rosemary N. Senguttuvan, Hyejin Cho, Xiwei Wu, Paul H. Frankel, Nora Ruel, Susan E. Yost, Mehdi Kebria, Ernest Han, Mihae Song, Maria de Leon, Marta Invernizzi, Melissa Eng, Raechelle Tinsley, Behrouz Salehian, Aimin Li, Daniel Schmolze, Sue Chang, Javier Arias-Stella, Thanh H. Dellinger

**Affiliations:** 1 Department of Surgery, City of Hope National Medical Center, Duarte, CA 91010, USA; rsenguttuvan@coh.org (R.N.S.); mkebria@coh.org (M.K.); ehan@coh.org (E.H.); misong@coh.org (M.S.); mdeleon@coh.org (M.d.L.); minvernizzi@coh.org (M.I.); 2 Integrative Genomics Core, City of Hope National Medical Center, Duarte, CA 91010, USA; hycho@coh.org (H.C.); xwu@coh.org (X.W.); 3 Department of Computation and Quantitative Medicine, City of Hope National Medical Center, Duarte, CA 91010, USA; pfrankel@coh.org (P.H.F.); nruel@coh.org (N.R.); 4 Department of Medical Oncology, City of Hope National Medical Center, Duarte, CA 91010, USA; suyost@coh.org; 5 Clinical Trials Office, City of Hope National Medical Center, Duarte, CA 91010, USA; meng@coh.org (M.E.); rtinsley@coh.org (R.T.); 6 Department of Endocrinology, City of Hope National Medical Center, Duarte, CA 91010, USA; bsalehian@coh.org; 7 Department of Pathology, City of Hope National Medical Center, Duarte, CA 91010, USA; aili@coh.org (A.L.); dschmolze@coh.org (D.S.); suchang@coh.org (S.C.); jariasstella@coh.org (J.A.-S.)

There was a mistake in the published paper [1] in Figure 1A (under “MA + PTE,” Pterostilbene 100 mg qD for 3 weeks, **“qD” should be “BID”**). This error was inadvertently missed by all authors/editors.

The same change was made in the Graphical Abstract, so the Graphical Abstract has also been updated.

**Figure 1:**
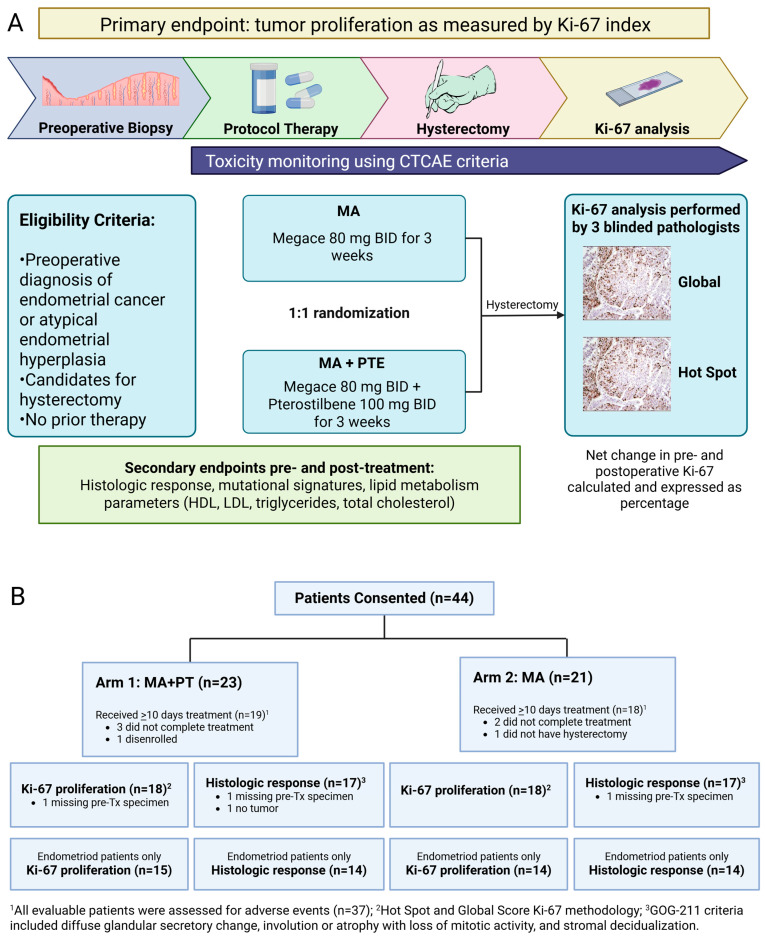
(**A**) Study schema of phase II randomized 2-arm study with primary endpoint Ki-67 index. Baseline endometrial biopsy was obtained as block or slides from initial diagnosis, or as new office endometrial biopsy. Intra-operative endometrial biopsy at time of hysterectomy was obtained if feasible. Post-treatment endometrial tumor was also obtained as block/slides if surgery was performed at a hospital outside of COH. (**B**) Flow diagram showing patients included in the study grouped by Arm 1 (*n* = 19) and Arm 2 (*n* = 18). Seven patients were not evaluable (5 received <10 days of treatment, 1 did not have hysterectomy, and 1 disenrolled). AE, adverse event; MA, megestrol acetate; PT, pterostilbene; Tx, treatment.

Under Section 2. Materials and Methods, Study design, the authors added the dosage of “100 mg twice a day” after “oral PT”. This error was inadvertently missed by all authors/editors.

The paragraph should be read as follows:

**Study Design:** The planned accrual for this study was 36 patients (Arm 1, *n* = 18 and Arm 2, *n* = 18). Patients were randomized in a 1:1 ratio to either Arm 1 (oral PT 100 mg twice a day + MA twice a day for three weeks), or Arm 2 (oral MA twice a day for three weeks). Therapy continued in the absence of unacceptable toxicity or progression of disease.

Under Section 3.5, Biomarkers of Response by IHC, “progression/regression” needs to be changed to “estrogen receptor/progesterone receptor.” This error was inadvertently missed by all authors/editors.

The paragraph was also updated.

*3.5. Biomarkers of Response by IHC*

Molecular markers of all eligible patients (*n* = 37) were analyzed at the beginning and completion of treatment to evaluate cell cycle regulation and cell survival (CDK4, Cyclin D1, BCL2, p-STAT3, and p-ERK1/2) and estrogen receptor/progesterone receptor (ER, PR). The results showed no significant differences between Arm 1 (*n* = 19) and Arm 2 (*n* = 18).

The correction has been approved by the Academic Editor. These changes do not affect the conclusions of the paper.
